# Dairy-Based Emulsions: Viscosity Affects Fat Difference Thresholds and Sweetness Perception

**DOI:** 10.3390/foods2040521

**Published:** 2013-11-27

**Authors:** Susann Zahn, Karin Hoppert, Franziska Ullrich, Harald Rohm

**Affiliations:** Institute of Food Technology and Bioprocess Engineering, Technische Universität Dresden, 01069 Dresden, Germany; E-Mails: susann.zahn@tu-dresden.de (S.Z.); karin.hoppert@tu-dresden.de (K.H.); f-ull@web.de (F.U.)

**Keywords:** difference threshold, just noticeable difference (JND), fat, sugar, viscosity, model emulsion

## Abstract

In complex emulsions, viscosity or viscosity-associated sensory attributes such as creaminess are important for quality assessment and product differentiation. Two sets of emulsions with fat or locust bean gum content being varied at seven levels were developed; the two emulsions at each level had similar apparent viscosity. Additionally, sugar concentration was kept constant either with respect to total emulsion, or with respect to the aqueous phase. Series of two-alternative forced choice tests were performed with one constant stimulus, and just noticeable differences were calculated using probability regression. The results show that, when viscosity was not compensated, it was easy for the subjects to (a) distinguish emulsions with different fat content when the fat content was addressed in the question, and to (b) distinguish emulsions with different fat or locust bean gum content when creaminess was addressed. For the latter descriptor, it is of minor importance whether viscosity is altered by fat content or a thickener. Weber fractions that were calculated for viscosity were approximately 0.20. The quantitative effects of viscosity on sweetness, however, depend on how product rheology was modified.

## 1. Introduction

Food emulsions are complex systems which consist of at least three constituents: two immiscible liquids (oil and water), and an emulsifier which reduces interfacial tension, prevents phase separation and which is, among others, responsible for kinetic stability. The main factors that affect the rheology and texture of emulsions are the viscosity of the continuous phase, the volume fraction of the disperse phase, droplet size distribution, and type and chemical properties of the emulsifier [1,2].

The adjustment of the rheological properties of emulsions to specific needs is important because that directly determines the usage quality. In many cases, additional ingredients (for example, hydrocolloids) are used to generate desired characteristics such as a static yield value, or an extended stability against creaming or sedimentation [3,4]. Apart from the changes in the rheological properties of the product and the resulting impact on sensory texture, it is also necessary to consider that the sensory perception of, for example, viscosity interacts with flavor and taste perception [3,5,6]. As regards texture, mainly properties that can be expressed by sensory descriptors such as mouthfeel or creaminess are of special importance in product development [7,8]. Another important issue in product design is to know how much the magnitude of a particular property may be altered (for example, by modifying the formulation) before the resulting changes can be perceived by the human, regardless whether she/he is an expert or represents the average consumer. In many cases, such a difference threshold (or just noticeable difference, JND) is directly related to the magnitude of a reference stimulus, usually denoted as Weber fraction [9,10].

In a previous study [11], we have shown for a model dairy system that, when directly addressing the fat content, it is difficult to distinguish low differences as long as the viscosity of the product is kept at a constant level. We have also shown that sweetness discrimination is, to some extent, affected by the fat content, even at similar emulsion viscosity. The aim of the present study was to evaluate the response of trained subjects towards differences in viscosity of model emulsions. For that purpose, two different systems where viscosity was pairwise adjusted by (a) fat content or (b) a hydrocolloid were developed and subjected to rheological and sensory analysis.

## 2. Materials and Methods

### 2.1. Materials

Raw materials for emulsion preparation were refined canola oil (Kaufland Warenhandels GmbH & Co KG, Neckarsulm, Germany), Viscogum GE locust bean gum (LBG; Cargill Texturizing Solutions, Hamburg, Germany), icing sugar (Pfeiffer & Langen GmbH & Co KG, Köln, Germany), a water-soluble butter-vanilla flavor (Dr. August Oetker KG, Bielefeld, Germany), β-carotene (Rudolf Wild GmbH & Co KG, Berlin, Germany), and skim milk powder (Käserei Champignon GmbH & Co KG, Heising, Germany).

### 2.2. Emulsion Preparation

Starting point was the formulation of Wendin & Hall [12], previously modified by Hoppert *et al.* [11] to ensure that emulsions with different fat content are perceived as iso-viscous and iso-sweet. In this study, the emulsions were prepared without adjusting viscosity. For that purpose, we mixed 16.5 g of an aqueous 3% (w/w) LBG dispersion with sugar, flavor, β-carotene, oil, 10% (w/w) reconstituted skim milk and water in the respective amounts (Table 1), and homogenized at 20,500 rpm for 15 min using a T15 ultra turrax (IKA Werke GmbH & Co KG, Staufen, Germany). Considering the lactose content of milk powder (51.8 g/100 g) and its relative sweetness of 0.33 [13], all emulsions had a sugar concentration in the aqueous phase, further denoted as sugar concentration index, of 0.05 (5%). The amount of added flavor was similarly adjusted to ensure a constant concentration in the aqueous phase. After production, the emulsions were stored at 5 °C and analyzed within 2 days.

Another set of seven emulsions was prepared at a constant fat content X_F_ = 1 g/100 g, and a constant skim milk content of 40 g/100 g; flavor and β-carotene were kept at 0.19 g/100g and 2.0 g/100 g, respectively. Compared to the formulations given in Table 1, sucrose and water addition had to be adjusted to achieve a total sugar concentration index of 0.047 (this corresponds to 0.050 when related to the aqueous phase; see first line of Table 1). Target was to create emulsions that had apparent viscosities η_a_ at 50/s and 1000/s that were close to η_a_ of the corresponding emulsions given in Table 1. This was achieved by increasing the LBG content from X_LBG_ = 0.5 g/100 g (equivalent to 16.5 g/100 g of a 3 % solution) on six additional levels.

In a third set of emulsions, the content of LBG, sucrose, flavor, β-carotene and skim milk was kept constant (see last line of Table 1); when X_F_ was lower than 29 g/100 g, the amount of incorporated water was increased to compensate for the missing mass. This procedure gives, regardless of X_F_, a total sugar concentration index of 0.033. Because of the different fat content, the sugar concentration related to the aqueous phase increased from 0.035 (1 g/100 g fat) to 0.050 (29 g/100 g fat).

**Table 1:** Formulation (g/100 g) of the emulsions with different fat content.

Oil	LBG ^1^	Sucrose	Flavor	β-Carotene ^2^	Skim milk ^3^	Water	Sugar concentration index (−)	
							In entire system ^4^	In aqueous phase ^5^
1.0	16.5	4.00	0.19	2.44	48.83	27.04	0.047	0.050
5.0	16.5	3.82	0.18	2.40	47.79	24.31	0.045	0.050
10.0	16.5	3.60	0.17	2.34	46.40	20.99	0.042	0.050
15.0	16.5	3.38	0.16	2.28	44.90	17.78	0.040	0.050
20.0	16.5	3.16	0.15	2.21	43.28	14.70	0.038	0.050
25.0	16.5	2.94	0.14	2.13	31.53	11.77	0.035	0.050
29.0	16.5	2.67	0.13	2.00	40.00	9.70	0.033	0.050

^1^ Aqueous solution of 3% (w/w) locust bean gum (LBG); ^2^ Aqueous solution of 1% (w/w) β-carotene, levels were adjusted to ensure similar color intensity; ^3^ Aqueous solution of 10% (w/w) skim milk powder; ^4^ Fraction of sugar (sucrose + 0.33 × lactose in skim milk); ^5^ Fraction of sugar (sucrose + 0.33 × lactose in skim milk) in the aqueous phase (sugar + water in LBG preparation + water in carotene + water in reconstituted skim milk considering skim milk moisture + additional water).

### 2.3. Rheological Measurements

Steady and dynamic rheological experiments were carried out using an MCR300 rheometer (Anton Paar Germany GmbH, Ostfildern, Germany) equipped with a CC27 concentric cylinder geometry. All measurements were performed after equilibrium at 9 °C in duplicate. Flow curves were recorded by increasing shear rate from 0.1/s to 1.000/s (10 points per decade) within 600 s. Small deformation properties were analyzed by strain sweeps where strain γ was increased from 0.001 to 10 at a constant angular frequency of 1 Hz, and by sweeping frequency from 0.1 to 10 Hz at γ = 0.005.

### 2.4. Sensory Evaluation

#### 2.4.1. General Conditions

Sensory analysis was performed in a laboratory with single booths at 22 ± 1 °C under red light. Ten milliliters samples were served on plastic spoons that came in petri dishes encoded with 3-digit random numbers. Tap water was provided *ad libitum* for mouth rinsing. The panel of fifteen that was recruited from students and PhD scientists of the institute was familiar with sensory investigations and had passed all of the standardized training procedures given in [14] with a score of at least 85%.

#### 2.4.2. Product Ranking

Depending on emulsion formulation, the assessors were asked to rank sets of four samples either with respect to fat content, creaminess, or sweetness. A balanced incomplete block design was established with fourteen panel members so that each of the seven emulsions was assessed eight times. The serving order of the samples within each set was randomized. Differences in the sums of the ranks that were assigned were evaluated using Page tests [15] and, additionally, by Friedman’s *F*-values. Multiple comparisons were performed by calculating the least significant difference.

#### 2.4.3. Difference Testing and JND Calculation

Difference thresholds for the sensory descriptors “fat content”, “creaminess” and “sweetness” were determined using sequences of two-alternative forced choice (AFC) tests. In each set, one sample was kept constant (reference), and the second sample was systematically varied. Per session, each of the fifteen panelists received four sample pairs in random configuration and was asked to pick the sample which was “higher”/“more intense” in the requested attribute. Each pair was evaluated twice by each panelist so that a total of 30 judgments were obtained. After plotting percentages of “higher”/“more intense” on probability ordinate against X_F_ or X_LBG_, just noticeable differences were calculated from regression functions as half of the difference between stimulus magnitudes that correspond to 25% and 75% correct identification [9,16].

#### 2.4.4. Estimation of Viscosity JNDs

**Figure 1:**
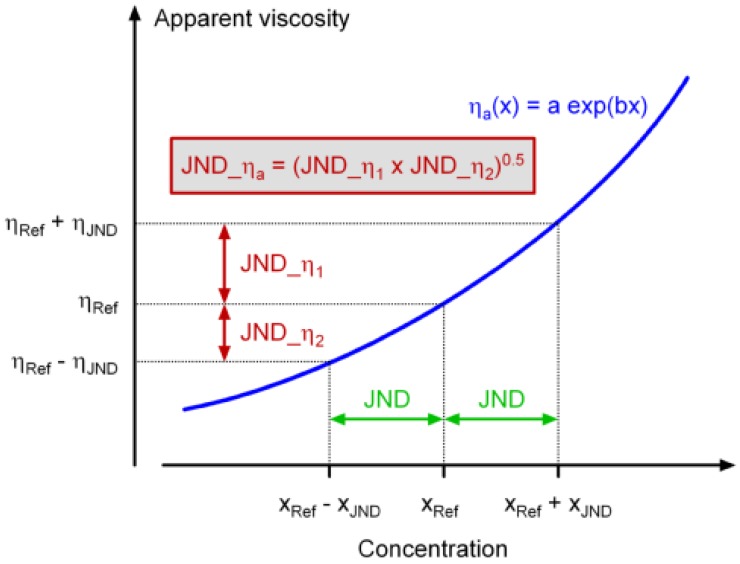
Schematic representation of the method for the calculation of just noticeable differences for viscosity (JND_η_a_).

Viscosity—concentration functions were used to calculate difference thresholds for apparent viscosity from JNDs that were obtained for fat content and creaminess (in the latter, the concentration parameter was X_LBG_). This transformation was based on the respective concentration dependent functions for η_a_ that was extracted from flow curves at 50/s. The viscosity JND was then calculated as geometric mean (Figure 1).

## 3. Results and Discussion

### 3.1. Viscosity Adjustment of Emulsions Using Locust Bean Gum

**Figure 2:**
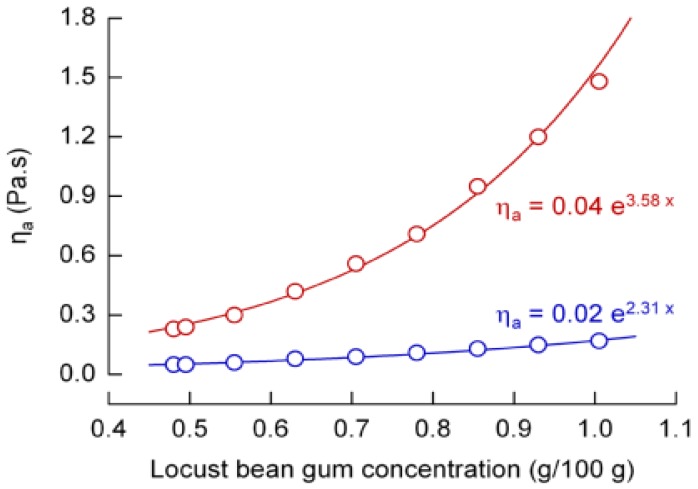
Apparent viscosity η_a_ as a function of locust bean gum concentration (data and respective fits) in emulsions with 1 g/100 g fat. Shear rate is 50/s (red) and 1000/s (blue).

For adjusting the viscosity of the seven emulsions with X_F_ = 1 g/100 g to that of the formulations given in Table 1, we first created systems where 0.02 g LBG was incorporated to mimic the viscosity effect of each g oil (*i.e.*, 0.1 g LBG to replace 5 g oil). Flow curves of these emulsions were recorded, and η_a_ taken at shear rates of 50/s and 1000/s showed an exponential relationship to X_LBG_ (Figure 2). The resulting viscosity equations (*R*^2^ > 0.98) were then solved with respect to X_LBG_. As two equations (for 50/s and 1000/s) were used, the final LBG concentration necessary to mimic a particular fat content was taken as the arithmetic mean of the two calculated values. The composition of the resulting emulsions is summarized in Table 2. All of these emulsions where viscosity was adjusted by LBG contained 1 g/100 g oil, 4.11 g/100 g sucrose, 0.19 g/100 g flavor, 2.0 g/100 g of 1% (w/w) aqueous β-carotene, and 40 g/100 g reconstituted skim milk (10% w/w), and the difference to a total of 100 g was made up with water. The sugar concentration index is constant at 0.05 in the entire system, and at 0.047 with respect to the aqueous phase.

**Figure 3:**
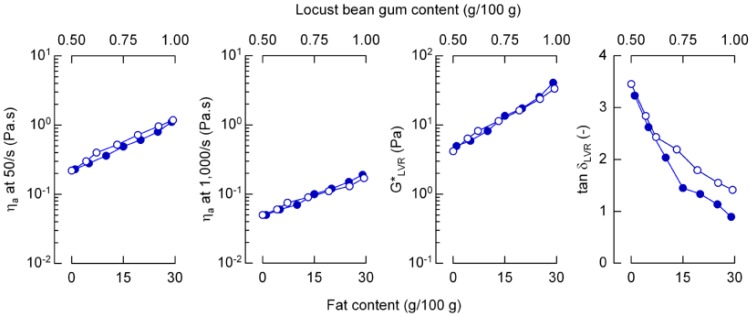
Apparent viscosity η_a_ at two shear rates, complex modulus G*_LVR_ and tan δ_LVR_ in the linear viscoelastic region of emulsions with different fat content (lower *x*-axis, closed symbols) and different locust bean gum content (upper *x*-axis, open symbols).

With the given formulations it was possible to use seven pairs of emulsions (line 1 *vs*. line 1, line 2 *vs*. line 2, *etc.* from Table 1 and Table 2) that were characterized by almost identical flow curves. Figure 3 shows η_a_ at two shear rates, and the complex modulus G* and tan δ in the linear viscoelastic region as a function of fat content (emulsions from Table 1) and LBG content (emulsions from Table 2). Generally, η_a_ at 50/s and 1000/s increased exponentially (*R*^2^ > 0.98) with increasing X_F_ or X_LBG_. The accordance in the shear rate range which is relevant during oral processing [17] allows comparing the results of sensory experiments that are obtained by using these emulsions. Apparent viscosity at 50/s can be expressed as a function of X_F_ by η_a_ = 0.21 exp (0.05 X_F_) (see Figure 1). Small deformation stiffness for the two emulsion sets was also in good agreement. Whereas, especially at higher concentration, fat droplets interact with each other at low strain, it is the entanglement of LBG at the given concentrations (which are above coil overlap concentration [18,19]) that mimic that behavior. The phase shift in the linear viscoelastic region was, however, different, especially at higher polymer concentration. The frequency at the cross-over of storage and loss modulus—the reciprocal of which refers to an indirect measure of the relaxation time [20]—was, especially for systems with a higher viscosity, lower for the emulsions where the fat content was varied (data not shown).

**Table 2:** Formulation (g/100 g) of the emulsions with constant fat content.

Oil	LBG ^1^	Sucrose	Flavor	β-Carotene ^2^	Skim milk ^3^	Water	Sugar concentration index (−)	
							In entire system ^4^	In aqueous phase ^5^
1.0	16.5	4.11	0.19	2.0	40.0	36.2	0.047	0.050
1.0	19.1	4.11	0.19	2.0	40.0	33.7	0.047	0.050
1.0	21.5	4.11	0.19	2.0	40.0	31.2	0.047	0.050
1.0	24.0	4.11	0.19	2.0	40.0	28.7	0.047	0.050
1.0	27.3	4.11	0.19	2.0	40.0	25.5	0.047	0.050
1.0	30.5	4.11	0.19	2.0	40.0	22.2	0.047	0.050
1.0	33.1	4.11	0.19	2.0	40.0	19.6	0.047	0.050

^1^ Aqueous solution of 3% (w/w) locust bean gum (LBG); ^2^ Aqueous solution of 1% (w/w) β-carotene; ^3^ Aqueous solution of 10% (w/w) skim milk powder; ^4^ Fraction of sugar (sucrose + 0.33 × lactose in skim milk); ^5^ Fraction of sugar (sucrose + 0.33 × lactose in skim milk) in the aqueous phase (sugar + water in LBG gel + water in carotene + water in reconstituted skim milk considering skim milk moisture).

### 3.2. Preliminary Sensory Tests

Three pairs of emulsions with corresponding viscosity (X_F_ = 5 g/100 g *vs*. X_LBG_ = 0.570 g/100 g; X_F_ = 15 g/100 g *vs*. X_LBG_ = 0.720 g/100 g; X_F_ = 25 g/100 g *vs*. X_LBG_ = 0.915 g/100 g; see lines 2, 4 and 6 in Table 1 and Table 2) were presented in two-AFCs to the panel in two replicate sessions. The results, expressed as the percentage of answers that were in accordance with the given sensory statement, and the respective significance levels [21] (Table 3) indicate that it was not possible to distinguish between the corresponding samples. It can also be concluded that the deviations in the rheological properties that were measured at low deformation (*i.e.*, in the linear viscoelastic region) are less relevant during the sensory assessment than shear viscosity.

**Table 3:** Percentages of coinciding statements in two-alternate force tests (*n* = 30) of three sample pairs with similar viscosity.

Sensory statement ^1^	Sample pair ^2^		
	F-5 *vs*. LBG-0.57	F-15 *vs*. LBG-0.72	F-25 *vs*. LBG-0.91
A has a higher fat content than B	50.0 ^3^	63.3 (*p* = 0.20)	63.3 (*p* = 0.20)
A is more creamy than B	43.3 (*p* = 0.58)	63.3 (*p* = 0.20)	43.3 (*p* = 0.58)
A is sweeter than B	43.3 (*p* = 0.58)	50.0	33.3 (*p* = 0.10)

^1^ A or B was assigned to the one or other sample randomly; ^2^ F-…, emulsions with given fat content (g/100 g) and 0.495 g/100 g locust bean gum (LBG); LBG-…, emulsions with given LBG content (g/100 g) and 1 g/100 g fat; ^3^ Number in brackets refer to probabilities (two-tailed). For (*n* = 30) and 50 % answers, no value is provided by Rössler *et al.* [21].

### 3.3. Sensory Evaluation of Emulsion Texture

**Figure 4:**
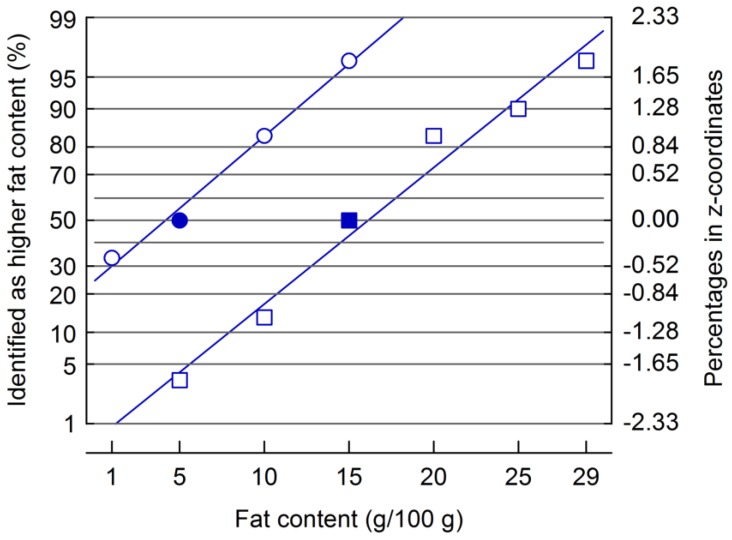
Plot of percentages of samples identified as being higher in fat content (left *y*-axis: percentage in normal probability grid, right *y*-axis: *z*-coordinates) as a function of emulsion fat content. Reference fat content (full symbols) was 5 g/100 g and 15 g/100 g.

Significant differences were obtained in the ranking of the emulsions from Table 1 with respect to the attribute “fat content”. The panel placed all samples in correct order, and the mean ranks ranged from 1.13 (X_F_ = 1 g/100 g) to 4.0 (X_F_ = 29 g/100 g). For this set of emulsions, the subsequent procedure with two-AFCs for threshold determination was carried out using two constant stimuli: X_F_ = 5 g/100 g, and X_F_ = 15 g/100 g. The JNDs that were calculated using the regression lines of the *z*-coordinates of correct answers *vs*. X_F_ (Figure 4) were 4.06 and 4.32 g/100 g fat, and the ratios of JND to constant stimulus magnitude (Weber fractions) were 0.81 and 0.29, respectively. These JNDs are much smaller than the approximately 14 g/100 g fat that were analyzed for emulsions with a fat content similar to that of this study, but that had a viscosity which was kept constant by using varying amounts of LBG [11]. The differences in the JND therefore indicate that it is obviously emulsion viscosity which, despite the question towards fat content in the sensory assessments, has been used by the panelists to find their decision.

Using the method illustrated in Figure 1, we subsequently calculated viscosity JNDs from those fat difference thresholds and, for η_a_ at a shear rate of 50/s, obtained 0.062 and 0.11 Pa.s at a reference X_F_ of 5 and 15 g/100 g. Because of the exponential relation between η_a_ and X_F_, the resulting Weber fractions are similar (0.22 and 0.24, respectively). This can be taken as an additional argument for suggesting viscosity-driven decisions during the sensory assessments. It is, however, not possible to compare these Weber fractions with viscosity differences as low as 5% that have been correctly identified in a recent study using triangle tests [22] because absolute viscosities were not specified there. The Weber fraction obtained by non-oral tactile viscosity two-AFC comparisons reported in literature is 0.19 [23].

In another set of ranking experiments, the emulsions with different LBG content at constant X_F_ = 1 g/100 g (see Table 2) were presented to the panel in a similar setup. Here, the panel members were free to select a descriptor that, in their individual opinion, best described between-samples intensity differences. Possible choices were: (a) mouthfeel, (b) viscosity, (c) creaminess, and (d) persistence in the mouth. The panel ranked the emulsions correctly (*i.e.*, by increasing order of LBG content or viscosity; see also Figure 3), with mean rank sums ranging from 1.0 (X_LBG_ = 0.50 g/kg) to 3.88 (X_LBG_ = 0.99 g/kg); the Page coefficient was 5.98 and significantly (*p* < 0.05) higher than the critical value of 1.64. Five panelists reported that they had used (d) for their decision, four panelists each decided on the basis of (b) and (c), and two panelists decided by mouthfeel. Based on the number of panelists that had used creaminess to distinguish between the emulsions, by additionally considering the opinion of the panel members regarding the general meaning of the presented descriptors (some of them had problems to interpret “persistence in the mouth”), and because creaminess is related to both fat content and viscosity in complex foods [7,24], we decided to use creaminess as descriptor in the subsequent two-AFCs for threshold determination.

The results of these experiments are summarized in Table 4. The correlation of the *z*-coordinates of the percentages of correct answers with X_F_ or X_LBG_ of the respective systems was significant (*R*^2^ > 0.89; *p* < 0.05). The JNDs obtained in the creaminess tests were, when expressed as fat content, similar for both references, but approximately 25% lower than the JNDs that were observed in the experiments where the decision was asked to be made with respect to fat content. This is presumably because creaminess, in contrast to fattiness, can be regarded as an attribute that is self-explaining and used more homogeneously, even by untrained panelists [25]. The JNDs that were calculated for LBG after analyzing emulsions with identical X_F_ and varying X_LBG_ were 0.07 g/100 g; this gives Weber fractions of 0.12 and 0.09, respectively. 

**Table 4:** Just noticeable differences for creaminess expressed as fat (JND_F_), locust bean gum (JND_LBG_) or viscosity (JND_η_), and corresponding Weber fractions K.

Reference (g/100 g)	Regression function ^1^	*R*^2^ (−)	JND_F_, JND_LBG_ (g/100 g)	K_F_ or K_LBG_ (−) ^2^	JND_η_ (Pa.s) ^3^	K_η_ (−) ^3^
*Emulsions with different fat content (Table 1)*						
X_F_ = 5	Y = 0.228X_F_ − 0.89	0.96	2.96	0.59	0.04	0.16
X_F_ = 15	Y = 0.213X_F_ − 3.10	0.91	3.16	0.21	0.08	0.17
*Emulsions with different locust bean gum content (Section 3.1.)*						
X_LBG_ = 0.57	Y = 10.16X_LBG_ − 5.75	0.89	0.07	0.12	0.07	0.22
X_LBG_ = 0.72	Y = 10.10X_LBG_ − 7.25	0.97	0.07	0.09	0.11	0.22

^1^ Y is percentage in z-coordinates; ^2^ k = JND/X; ^3^ Calculated using the schematic presentation in Figure 1.

The JNDs that were finally calculated from the corresponding apparent viscosity at a shear rate of 50/s were related to the reference stimuli in a way so that the resulting Weber fractions were 0.16 (emulsions with constant X_LBG_ and varying X_F_) and 0.22 (constant X_F_, but varying X_LBG_). Rheological properties at small deformation, especially the higher ratio of viscous to elastic contributions in emulsions with a higher X_LBG_ (see Figure 3), may serve as a possible explanation for Weber fraction differences: results of multivariate analysis showed that a higher sensory creaminess is apparently linked to a lower loss tangent at small deformations for comparable systems [26]. Emulsions with varying fat content exhibit a lower tan δ and, as indicated by the viscosity related Weber fraction, can be distinguished more easily.

### 3.4. Sweetness Difference Thresholds as Affected by Emulsion Viscosity

**Figure 5:**
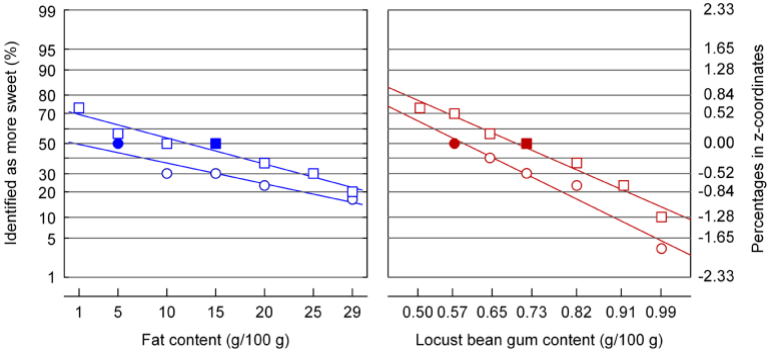
Judgment of sweetness of emulsions with different viscosity. Viscosity was adjusted by using different levels of fat or locust bean gum. The fraction of sugar in the aqueous phase was 0.05. Reference samples (full symbols) contained 5 or 15 g/100 g fat, or 0.57 or 0.72 g/100 g locust bean gum.

The formulations given in Table 1 (different fat content) and the systems where viscosity was adjusted with LBG (see Table 2) have a constant sugar concentration in the aqueous phase; such systems have been shown as being iso-sweet in emulsions with similar viscosity [11]. Initial ranking tests that were performed with respect to sweetness revealed, however, that the systems with higher X_F_ or X_LBG_ were perceived as significantly less sweet when viscosity was not adjusted. This is in line with cross-modal interactions reported in studies that refer to textured samples, e.g., [4,6,27], or even to low-viscosity foods [28]. At varying fat content, the mean ranks ranged between 3.38 (highest sweetness at X_F_ = 1 g/100 g, η_a_ (50/s) = 0.23 Pa.s) and 1.63 (lowest sweetness at X_F_ = 29 g/100 g, η_a_ (50/s) = 1.11 Pa.s). Mean sweetness ranks for samples with varying X_LBG_ ranged between 3.13 (low viscosity, low X_LBG_) and 1.88 (high viscosity, high X_LBG_). Figure 5 shows the results of the two-AFCs for the two emulsion types as a function of fat content or locust bean gum content (for apparent viscosity of these systems, see Figure 3), which confirm the results of the ranking experiments. Two possible mechanisms can be mentioned in context with these findings. It has been shown that the liberation of saliva during oral manipulation of systems with a higher viscosity [4,29] is higher so that concentration differences could be partly attenuated or compensated. A second explanation is related to the fact that, even at a constant X_LBG_ in the emulsions with different fat content, the amount of LBG in the aqueous phase varied from 0.54 to 0.74 g/100 g. That concentration is above coil overlap concentration [18,19]; at such concentrations it has been shown that polymeric carbohydrates results in a reduced intensity of taste and flavor perception [3,5]. The fact that the slope for sweetness reduction in LBG thickened systems is steeper might point on differences in the suppressing mechanisms; using different systems and methods, Hollowood *et al.* [30] showed comparable results. However, the statement that texture modification may compensate for a loss of sugar-induced sweet taste intensity because of the additive relationship that has been addressed for gel-like systems [31] is not supported.

An effect of emulsion viscosity on perceived sweetness was also observed after subjecting samples to two-AFCs that had a total sugar concentration index of 0.033; in the samples of set 3, the sugar content related to the aqueous phase varied between 0.035 (for X_F_ = 1 g/100 g fat) and 0.05 (for X_F_ = 29 g/100 g fat). Despite similar total sugar, the samples with lower sugar content in the aqueous phase (*i.e.*, samples with higher viscosity) were judged as significantly less sweet. This indicates that, when considering different amounts of the disperse phase in a particular emulsion, when considering different amounts of thickeners, or when considering different system viscosity, it is absolutely necessary to ensure experimentally that no undesired side effects which result from improper formulation are present.

## 4. Conclusions

The difference thresholds for fat content obtained in this study were much lower than the thresholds that were analyzed by subjecting iso-viscous emulsions to sensory analysis. This indicates that, even when fat content is the given judgment attribute, subjects tend to judge such an attribute by a descriptor that is associated with fat content (here, apparent viscosity). For two sample sets with either varying fat content and constant thickener, or varying thickener content and constant fat, creaminess thresholds that were transferred into units of apparent viscosity resulted in Weber fractions of approximately 0.20, that correspond well with data from non-oral viscosity assessment. Sweetness judgments of emulsions with different viscosity indicate that interactions with viscosity have to be considered, but also that it is important by which means a viscosity modification is approached. The interactions can be provoked by changing fat content, but also by adjusting viscosity using a polymeric carbohydrate thickener; quantitative differences in the action on sweetness should, however, be considered.
